# Reply to: How carvedilol does not activate β_2_-adrenoceptors

**DOI:** 10.1038/s41467-023-42849-4

**Published:** 2023-11-30

**Authors:** Evi Kostenis, Jesus Gomeza, Elke Miess-Tanneberg, Nina Kathleen Blum, Tobias Benkel, Andy Chevigné, Carsten Hoffmann, Peter Kolb, Viacheslav Nikolaev, Maria Waldhoer, Martyna Szpakowska, Asuka Inoue, Stefan Schulz

**Affiliations:** 1 Molecular, Cellular, and Pharmacobiology Section, Institute of Pharmaceutical Biology, University of Bonn, 53115 Bonn, Germany; 2 Institute of Pharmacology and Toxicology, Jena University Hospital, Friedrich-Schiller-University of Jena, 07747 Jena, Germany; 3 Immuno-Pharmacology and Interactomics, Department of Infection and Immunity, Luxembourg Institute of Health (LIH), L-4354 Esch-sur-Alzette, Luxembourg; 4 Institute for Molecular Cell Biology, CMB-Center for Molecular Biomedicine, Jena University Hospital, Friedrich Schiller University of Jena, 07745 Jena, Germany; 5 Department of Pharmaceutical Chemistry, Philipps-University of Marburg, 35032 Marburg, Germany; 6 Institute of Experimental Cardiovascular Research, University Medical Center Hamburg-Eppendorf, 20246 Hamburg, Germany; 7 InterAx Biotech AG, 5234 Villigen, Switzerland; 8 Graduate School of Pharmaceutical Science, Tohoku University, Sendai, 980-8578 Japan; 9 Present Address: ISAR Bioscience, Semmelweisstraße 5, 82152 Planegg, Germany; 10 Present Address: Ikherma Consulting Ltd, Hitchin, SG4 0TY UK

**Keywords:** G protein-coupled receptors, Cell signalling

**replying to** R.J. Lefkowitz et al. *Nature Communications* 10.1038/s41467-023-42848-5 (2023)

We thank Dr. Robert Lefkowitz and co-authors for their comments on our article “How carvedilol activates β_2_-adrenoceptors”^1^. In their critique^2^, they state that our study concludes that intrinsic sympathomimetic activity (ISA) is the mechanism responsible for the beneficial actions of carvedilol and that this is incorrect. First and foremost, we neither concluded definitively that ISA explains the superior clinical profile of carvedilol nor do we suggest any changes to the well-established, beneficial clinical practices. We did not intend to make inferences between the mechanism of carvedilol action and its clinical significance. Our statements considering any potential in vivo and clinical implications of our work were purely speculative and meant to stimulate further research and discussion. The pertinent question in Benkel et al.^1^ is rather to illuminate how carvedilol, a widely used beta blocker that works by inhibiting β_1_-ARs, activates cellular signaling via the β_2_-AR subtype. Specifically, we state in the abstract “beyond blockade of β_1_-ARs, arrestin-biased signaling via β_2_-ARs is a molecular mechanism proposed to explain the survival benefits. Here, we offer an alternative mechanism to rationalize carvedilol’s cellular signaling”.

Herein, we can only reiterate that we established novel and recapitulated previously used cellular systems to bring about carvedilol signaling through β_2_-ARs and find that G-proteins but not arrestins initiate all detectable cellular signals in these conditions. Others postulate arrestin-biased agonism for carvedilol at the β_2_-AR^2–4^ and hence a debate is sparked on how precisely carvedilol activates this β-adrenoceptor subtype. We certainly stand by all conclusions presented in our published work^1^, and we explain below how a thorough comparison of the data contained in Benkel et al.^1^ with those referenced by Lefkowitz et al.^2^ along with enhanced semantic precision will help resolve the apparent confusion and provide a unified explanation of events.

## The ISA of carvedilol—clinical implications

We are keen to stress that the molecular mechanism underlying carvedilol activation of the β_2_-AR in engineered cells and even in primary neonatal cardiomyocytes must not be confounded with the clinical relevance and value of β-blockers that may or may not display varying degrees of ISA. ISA was originally coined to describe the ability of β-blockers to also stimulate β-ARs to some extent and this partial activation may become manifest as elevation of cAMP, heart rate, or force of contraction^5–7^. As pointed out by Lefkowitz and co-authors^2^, there is clinical consensus from a number of post-myocardial infarction trials carried out in the 1970’s and 1980’s that β-blockers with too much ISA are not beneficial and may even increase mortality of heart failure patients^2^, in apparent contrast to the only study cited in Benkel et al.^1^. However, Lefkowitz et al.^2^ do not mention that the study cited by Benkel et al.^1^ was published in 1990 and so made reference to several of these earlier trials as well as unveiled that a subgroup of high-risk patients not enrolled in these trials did indeed benefit from mild ISA^8,9^. Thus, whether ISA may be beneficial or not is likely dependent on the degree of ISA, the selected patient population, and, finally, the involved β-receptor subtype.

In Benkel et al.^1^ we use the term ISA to refer specifically to the weak activation induced by carvedilol through the β_2_-AR (note that the detrimental effects of ISA are associated with the β_1_-AR), and we employed cellular conditions that allow both detection of this weak activation and its mechanistic dissection. For the above reasons, our discussion on the clinical significance of ISA in Benkel et al.^1^ is short and entirely speculative. Clinical implications were not the focus of our work.

## The ISA of carvedilol—variability and context dependence

Lefkowitz et al.^2^ correctly point out that carvedilol stimulation of β-ARs is variably detected across species and experimental paradigms. For instance, in untransfected HEK293 cells which express low (physiologic) levels of β_2_-AR, carvedilol neither elevates cAMP nor stimulates phosphorylation of ERK/MAP kinases. It follows that overexpression systems are clearly demanded to study the low efficacy carvedilol signaling in the HEK cellular context. We appreciate the comment^2^ that “carvedilol does indeed promote some very low level of cAMP accumulation provided that receptor levels are raised dramatically by overexpression and that high concentrations of carvedilol are used”. In fact, the authors state that “The concentrations of carvedilol used in Benkel’s overexpressing cells, 10 micromolar for many studies, and above one micromolar in most, are well above the plasma levels the drug ever achieves clinically.” We are well aware that elevation of cAMP but also phosphorylation of ERK/MAP kinases (pERK), require such high carvedilol concentrations as well as overexpressing cells. The same high concentration of carvedilol, 10 micromolar, and β_2_-AR overexpressing cells was used in the paper of the Lefkowitz group (Wisler et al.^3^) to coin the unique mechanism of carvedilol action: stimulation of β-arrestin signaling. Given the notion that carvedilol stimulation of low-level functional responses in HEK293 cells requires β_2_-AR overexpression, it is even more surprising that some very recent investigations, referenced in ref. ^2^, study cAMP production at endogenous β_2_-AR expression but pERK in β_2_-AR overexpressing cells^10^. This clearly highlights the need for a very careful choice of experimental setup when designing studies to investigate biased signaling, as well as a careful interpretation and discussion of the results obtained from such experiments.

Low efficacy β_2_-AR activation by carvedilol is not only observable by us and others in recombinant cells (HEK293, CHO-K1) overexpressing the β_2_-AR^1,11,12^, but also in embryonic cardiomyocytes with endogenous β_2_-AR expression^1^, and even in explants from human ventricular myocardium^7^. We appreciate the comment of Lefkowitz and colleagues that the ISA of carvedilol is detectable in only one out of seven human explants, clearly attesting to both (i) its predominant action as β-blocker and (ii) the ability to also activate β-receptors to some extent. Given that low efficacy β_2_-AR activation is quite variably detected for carvedilol^1,2,6,7,10–13^, it is very likely cellular context-, patient- and disease-dependent. In our neonatal cardiomyocyte preparation, we recorded very slow and low-level cAMP elevation (Fig. 3 in Benkel et al.^1^), but carvedilol enhancement of spontaneous cardiomyocyte beating was only observable in cells with spontaneous beating ≤60 bpm (as stated in the legend), and after blockade of β_1_-ARs with the β_1_-preferring blocker CGP-20712A (Fig. 3h in Benkel et al.^1^). Thus, our experimental setup is distinct from many other studies that did not observe ISA in the endogenous context, as we deliberately needed to create cellular conditions that are permissive for detection of low-level β_2_-AR activation. Whether low-level β_2_-AR activation or any other mechanistic attribute is responsible for the beneficial actions of carvedilol in the clinic is clearly beyond our discretion.

## G-protein signaling versus arrestin signaling

In Benkel et al.^1^, we chose to use engineered HEK293 cells, depleted by CRISPR/Cas9 of either G-proteins or β-arrestins, to investigate the molecular details underlying the low-level carvedilol signaling. Because HEK293 cells have been used to coin the unique mechanism of carvedilol signaling^3^, and because such cells are by far more amenable than primary cells to genetic manipulations and to the identification of the involved signaling components, engineered HEK293 cells are the line of choice to disentangle why apparently comparable experimental settings may lead to such disparate mechanistic conclusions: G-protein versus arrestin signaling.

We believe that a thorough look at experimental details and original data obtained in such engineered cells is likely to provide a plausible explanation. While Benkel et al.^1^ determined cAMP elevation and phosphorylation of ERK/MAP kinases in HEK293 cells overexpressing the β_2_-AR and lacking either G-proteins or arrestins, Lefkowitz et al.^2^ and the few additional studies referenced therein only used arrestin-depleted cells but did not take advantage of the G-protein-depleted counterparts. We believe this is necessary for two reasons: first, arrestin-depleted cells only allow to investigate arrestin contribution to G-protein-driven signals; second, G-protein-depleted cells are a prerequisite to visualize the suggested arrestin transducer function, i.e., the ability of arrestins to promote signaling in their own right and independent of functional G-proteins. If this is not done, mechanistic conclusions on a signaling driver role of arrestins must not be drawn and may likely be erroneous and misleading.

Looking ahead, enhanced semantic precision will also be indispensable to solving the problem. The term “arrestin signaling” was coined to reflect the independent transducer function of this protein family and to differentiate the arrestin pathway from the canonical G-protein-mediated signal transduction. However, the very same term, arrestin signaling, is also used to denote the arrestin contribution to G-protein-initiated signal transduction, which is mechanistically distinct. Indeed, arrestin contribution to G-protein-initiated signaling explains both diminished pERK upon reduction of βarr1/2 expression and enhanced pERK1/2 in βarr1/2 KO cells upon βarr1/2 re-expression after GPCR stimulation^4^. However, to truly unmask the arrestin transducer function, experimental paradigms would require genetic deletion or pharmacological inhibition of heterotrimeric G-proteins and β-arrestins. Unfortunately, studies like these are still scarce^1,14–18^, although they are now technically feasible due to the advent of gene editing technologies yielding mammalian expression hosts with either targeted deletion of the α subunits of the four G-protein classes^14,18^ or arrestins^14–16,18^. Along with G-protein class-specific inhibitors such as pertussis toxin, FR900359, and YM254890^19–21^, as well as naturally arrestin-biased receptors that internalize but do not signal through heterotrimeric G-proteins^22^, these engineered cells offer a widespread and useful approach to discriminate, unambiguously, transducers of receptor signals from proteins that assist but do not drive signaling on their own. The collective experimental evidence gathered with such genome-edited cells nicely illustrates that carvedilol used at high concentrations in β_2_-AR overexpressing cells^1–4^ actively promotes signaling in a process that is G-protein-dependent and arrestin-assisted.

Controversy exists in every field of science and is essential to spur new ideas and scientific progress. This communication focuses on the controversy surrounding the molecular mechanism that underlies low efficacy carvedilol activation of the β_2_-AR. The reason why carvedilol shows a superior profile in clinical practice remains entirely speculative and needs to take into account all properties of this β-blocker, including but not limited to its interaction with α-adrenergic receptors^23^.
